# Interindividual Variability in Postprandial Plasma Fructose Patterns in Adults

**DOI:** 10.3390/nu16183079

**Published:** 2024-09-13

**Authors:** Mia Gladding, Xiaotao Shen, Michael P. Snyder, Peter J. Havel, Sean H. Adams

**Affiliations:** 1 Department of Food Science and Nutrition, California Polytechnic University, San Luis Obispo, CA 93407, USA; mgladdin@calpoly.edu; 2 Department of Genetics, School of Medicine, Stanford University, Stanford, CA 94306, USA; shenxt@stanford.edu (X.S.); mpsnyder@stanford.edu (M.P.S.); 3 Stanford Center for Genomics and Personalized Medicine, Stanford University, Stanford, CA 94306, USA; 4 Department of Molecular Biosciences, School of Veterinary Medicine, University of California, Davis, CA 95616, USA; pjhavel@ucdavis.edu; 5 Department of Nutrition, University of California, Davis, CA 95616, USA; 6 Department of Surgery, University of California Davis School of Medicine, Sacramento, CA 95817, USA; 7 Center for Alimentary and Metabolic Science, University of California Davis School of Medicine, Sacramento, CA 95817, USA

**Keywords:** polyol, diabetes, GLUT5, precision nutrition, personalized nutrition

## Abstract

High fructose consumption is associated with an increased risk of cardiometabolic disease, and fructose feeding dose-dependently induces markers reflective of poor metabolic health. However, unlike glucose, surprisingly little is known about person-to-person differences in postprandial plasma fructose patterns. Herein, we performed post hoc analyses of two published studies to address this question. In the first cohort, 16 participants’ all-day plasma fructose concentration patterns (08:00–23:30) were determined (8 women and 8 men) while consuming mixed meals (breakfast, lunch, and dinner) with a fructose-sweetened beverage at each meal (30% of calories). Individually plotted results demonstrate remarkably disparate fructose patterns with respect to peak concentration and timing. A secondary study confirmed substantial interindividual variability in plasma fructose patterns over 240 min in 16 adults consuming Ensure^®^, a commercially available mixed macronutrient drink containing a low dose of fructose. The health ramifications of interindividual variations in postprandial fructose metabolism and the underlying physiological mechanisms driving differences in post-meal blood patterns remain to be explored. Future research is warranted to determine if interindividual variability in fructose digestion, metabolism, and postprandial blood concentration patterns is associated with cardiometabolic health phenotypes and disease risk.

## 1. Introduction

The typical American diet pattern is characterized by a low healthy eating index, driven in part by higher than recommended levels of added sugars [1]. Higher intakes of added sugars (i.e., sugar-sweetened beverages) in adults and pediatric populations have been associated with an increased risk of cardiometabolic dysregulation, including obesity, nonalcoholic fatty liver disease (NAFLD), cardiovascular disease, or type 2 diabetes (see, e.g., [2,3,4]). There were dose-responsive increases in fasting and postprandial triglycerides, LDL cholesterol, apo-lipoproteins B and C3, liver fat content, and uric acid and lowered insulin sensitivity during 2 weeks of consuming beverages containing high-fructose corn syrup at 10%, 17.5%, or 25% of daily calories along with mixed meals in obese adults [5,6]. At a population level, the U.S. Dietary Guidelines for Americans and the World Health Organization recommend that dietary patterns should include no more than 10% of calories as added sugars, typically consumed in the forms of sucrose (50% fructose), high-fructose corn syrup (~55% fructose), or other sugars such as the glucose precursor maltodextrin. Despite these recommendations, the majority of the U.S. population does not achieve this goal. For instance, a recent study examining a cross-section of 2154 adults included in the U.S. National Health and Nutrition Examination Survey (NHANES) found that just ~31% of the population consumes < 10%, ~32% consumes 10–15%, and ~37% consumes > 15% of their daily calories in the form of added sugars [7].

The precise amounts and contexts that increase cardiometabolic disease risk in response to dietary sugars remain to be fully elaborated. By their nature, most epidemiological studies or meta-analyses utilize odds ratio (OR) or risk ratio (RR) approaches applied with a public health lens; however, this may not fully capture the risk at the individual level. In fact, it is underappreciated that most persons studied in sugar-related disease risk epidemiology studies or meta-analyses retain a cardiometabolic disease risk profile similar to that of low sugar consuming controls. Thus, it is possible that regular fructose or sugar consumption per se does not inevitably lead to cardiometabolic disease for all individuals, indicative of person-to-person variability in susceptibility. The reasons for variable outcomes are not known, and knowledge related to interindividual differences in postprandial fructose metabolism and circulating fructose excursions would be useful to attain.

Differences in fructose absorption and transport may have significant effects on the variability in hepatic fructose metabolism and postprandial circulating fructose concentrations. The intestinal, hepatic, and tissue metabolisms of fructose have been reviewed in detail [8,9,10,11,12]. Unlike the insulin-regulated tissue metabolism of glucose, hepatic fructose uptake and metabolism are relatively unregulated and mostly dependent on fructose uptake from the intestine. After entry into the liver via the portal vein, most fructose is converted to fructose-1-phosphate (F1P) by fructokinase, and then aldolase B converts F1P to phosphotriose—this promotes robust synthesis of lactate and fatty acids (in the intestine and liver, packaged into triglycerides) when fructose-containing sugars are consumed in significant amounts. The process of fructose metabolism through F1P synthesis is thought to increase levels of uric acid via ATP utilization and coincident generation of AMP; the latter’s degradation leads to enhanced inosine monophosphate and uric acid synthesis [3]. The liver is the primary site of uptake from the portal circulation [13,14,15,16,17,18,19,20], but significant fructose metabolism also occurs in the small intestine (see [11]). The uptake of fructose in the small intestine is optimized in the presence of glucose, facilitated through GLUT5; in fact, high levels of fructose intake in isolation can sometimes cause gastrointestinal (GI) discomfort due to malabsorption. The small intestine converts a portion of fructose to glucose, organic acids, and chylomicron triglycerides [9,21,22]. Other sites of fructose uptake that could influence peripheral blood fructose levels include the kidney [23], skeletal muscle during exercise [24], and possibly adipose tissue (since there is significant fructose uptake and metabolism in rat adipocytes; [25,26,27,28]).

Measurements of postprandial concentrations of plasma fructose may reveal unique patterns that are relevant to the effects of fructose on cardiometabolic health. It is well established that fasting and postprandial plasma glucose concentrations can identify individual diabetes and pre-diabetes phenotypes. In addition to routine oral glucose tolerance tests, there is growing interest in monitoring glucose during mixed macronutrient settings that may be more reflective of typical postprandial conditions. For instance, Zeevi et al. studied blood glucose in an 800-person cohort in response to glucose-containing foods [29], and found remarkable variability in glycemic responses in terms of magnitude and patterns after consuming standardized foods. Interindividual differences following a mixed macronutrient tolerance test have also been used to define variability in the uptake and metabolism of specific nutrients, primarily with a focus on lipids or glucose [30]. Newman et al. highlighted the utility of characterizing the person-to-person variability in circulating triglycerides, glucose, and insulin sensitivity through a mixed macronutrient tolerance test [31]. As with glucose, a better understanding of individual variability in fructose metabolism following an oral fructose challenge could potentially support the development of improved personalized nutrition and public health strategies that consider dose vs. disease risk outcomes in individuals or population subsets. To this end, we have characterized the interindividual differences in postprandial plasma fructose concentrations in women and men, characterizing all-day plasma fructose patterns across individuals under highly controlled feeding conditions in which a relatively high dose of fructose was administered in sweetened beverages consumed with breakfast, lunch, and dinner. The study is a post hoc analysis of individual participant data derived from a cohort presented previously [32]. In a secondary study, we assessed acute plasma fructose concentrations across individuals from a recent study in which a commercially available, low-fructose, mixed macronutrient drink was consumed [33]. We hypothesized that like glucose and many other nutrients, there would be substantial person-to-person variability in postprandial plasma fructose patterns. Our working model proposes that—at least at higher fructose intakes—variable postprandial plasma fructose patterns in part reflect differences in fructose absorption, hepatic fructose uptake, and fructose-associated de novo lipogenesis. 

## 2. Methods

### 2.1. Study Populations and Feeding Paradigms 

**Cohort 1 (primary study):** Relevant details regarding the participants and the process of collecting data are outlined here briefly, but further detail can be found in the original paper by Teff et al. [32]. The volunteers for that primary study consisted of 17 adults with obesity, with a body mass index (BMI) range of 30–38. There were 8 women who ranged from 18–36 years of age and 9 men who ranged from 25–49 years of age. After the initial interview and providing informed consent, subjects were given a physical examination at the Clinical and Translational Research Center of the University of Pennsylvania. The study was approved by the Committees on Studies Involving Human Subjects at the University of Pennsylvania (Philadelphia, PA, USA) and the Institutional Review Board at the University of California, Davis (Davis, CA, USA). The volunteers underwent physical examinations including an electrocardiogram and a thorough medical history to confirm that there were no pre-existing chronic illnesses, including diabetes. The only medication allowed among the subjects included hormonal birth control. The inclusion criteria were fasting triglyceride concentrations < 200 mg/dL, fasting glucose < 90 mg/dL, hemoglobin ≥ 12 g/dL, and blood pressure < 150/99 mmHg. Women were screened for pregnancy and were only included in the study if the pregnancy test was negative. One male subject dropped out of the study without completing both trials and his data were not included in the results.

Participants were provided with identical meals, with the only difference being the number of calories provided based on the subjects’ estimated energy needs (Mifflin St. Jeor with activity factor of 1.2). Subjects received 25% of their daily energy requirement from breakfast, 35% from lunch, and 40% from dinner. The food provided for breakfast consisted of a bagel with cream cheese and scrambled eggs at 0900 h. Lunch was served at 1300 h and consisted of a chef’s salad with turkey, cheese, and a dinner roll. Dinner, served at 1800 h, consisted of chicken breast, mashed potatoes, carrots, and a buttered roll. More details related to the meal compositions and conditions are described by Teff et al. [32]. The total calories within the meals were provided as 30% of the energy from fat, 55% from carbohydrates (25% complex carbohydrates, and 30% from fructose-sweetened beverages), and 15% from protein. The meals were consumed with the addition of a fructose-sweetened beverage, prepared as follows: fructose (15%, weight/weight) was dissolved in an unsweetened drink mix. As an illustration, using a 2500 kcal/day energy requirement, the breakfast, lunch, and dinner would have provided fructose at 47, 66, and 75 g, respectively. Longitudinal blood samples were collected over 24 h; baseline blood samples were drawn at 0800, 0830, and 0900 h before the first meal was consumed, and then, 33 additional samples were collected either 30 or 60 min apart. Plasma fructose was measured biochemically with a modification to a food fructose kit (Megazyme, catalog item K-FRUGL; Xygen Diagnostics Inc., Burgessville, ON, Canada), as described previously [34].

**Cohort 2:** We also had an opportunity to consider results from a recent “multi-omics” phenotyping study [33] that examined plasma metabolomics patterns after consuming a mixed macronutrient drink containing a much lower dose of fructose compared to Cohort 1. In this secondary study, a post hoc analysis was conducted to examine plasma fructose results derived from mass spectrometry analysis over 240 min postprandially in 9 women and 7 men for whom fructose results were available. Details related to the original full cohort demographics and the methods including metabolomics analyses are described in the original paper [33]. Briefly, metabolites were formally identified by matching fragmentation spectra and retention time to analytical grade standards, when possible (Level 1), or by matching experimental tandem mass spectrometry results to fragmentation spectra in publicly available databases (Level 2) using metID. The Metabolomics Standards Initiative parameters were used for metabolite annotation confidence [35]. Fructose was identified using the MONA database based on accurate mass and the MS/MS (MS^2^) spectral data.

Following an overnight fast, participants consumed two Ensure^®^ Original nutrition shakes (Abbott). This mixed macronutrient challenge contained 440 kcal consisting of 64 g of carbohydrate, 18 g of protein, and 12 g of total fat. Of the carbohydrates, 18 g were added sugars including corn maltodextrin (a source of glucose upon digestion) and sucrose (yielding glucose plus fructose upon digestion). Specific amounts of ingredients are proprietary and not publicly available, but the dose of fructose would have been just a fraction of that provided in the primary study (Cohort 1). For example, under the assumption that the 18 g of added sugars was split evenly between 9 g of maltodextrin and 9 g of sucrose, then fructose would represent 4.5 g in the shake. The ingredients as listed by the vendor are as follows: water, corn maltodextrin, sugar, milk protein concentrate, a blend of vegetable oils (canola, corn), soy protein isolate, and nonfat milk. There is less than 0.5% of vitamins and minerals (potassium citrate, magnesium phosphate, calcium carbonate, sodium citrate, ascorbic acid, choline chloride, calcium phosphate, potassium chloride, potassium hydroxide, ferrous sulfate, dl-alpha-tocopheryl acetate, zinc sulfate, niacinamide, calcium pantothenate, magnesium sulfate, pyridoxine hydrochloride, thiamine hydrochloride, copper sulfate, riboflavin, vitamin A palmitate, folic acid, potassium iodide, chromium chloride, sodium selenate, sodium molybdate, phylloquinone, biotin, vitamin D3, vitamin B12), natural and artificial flavors, cellulose gel, salt, cellulose gum, monoglycerides, soy lecithin, carrageenan, and sucralose.

### 2.2. Statistics

Our study design and result evaluation align with an “n of 1” approach in which individual plots of metabolite values are provided. To evaluate the statistical significance of plasma fructose over time in mean plasma fructose values in Cohort 2, a one-way analysis of variance (ANOVA) was applied (GraphPad Prism 9, GraphPad, San Diego, CA, USA).

## 3. Results

**Figure 1:**
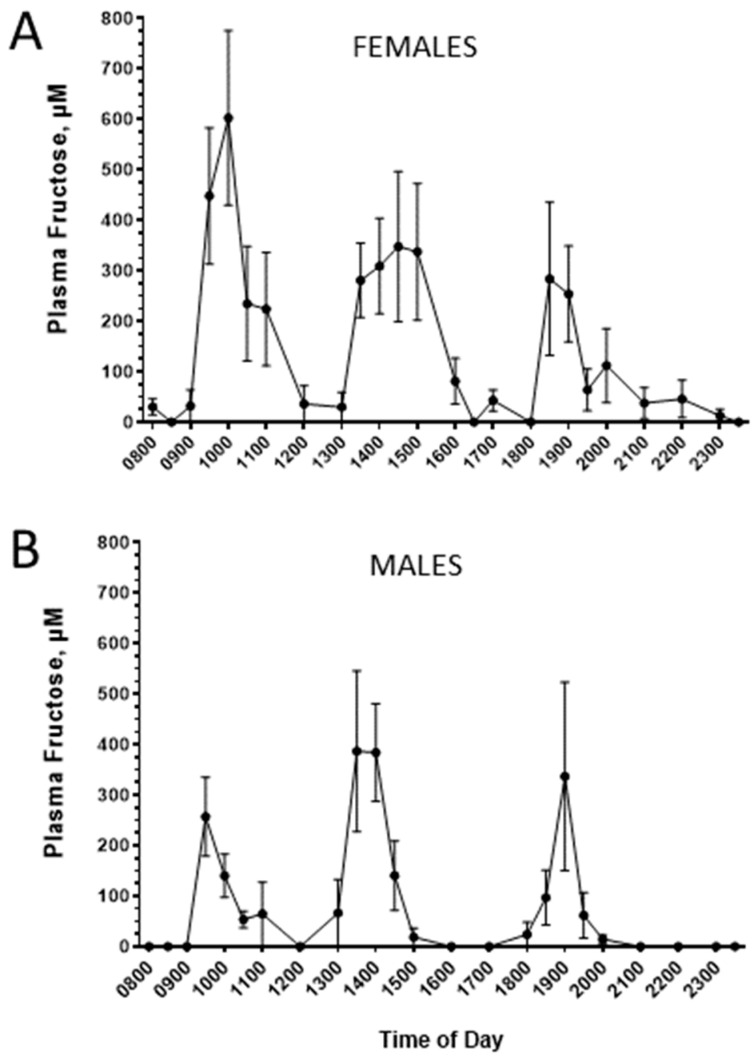
The patterns of plasma fructose in (**A**) women and (**B**) men following breakfast, lunch, and dinner (each including a fructose-sweetened beverage). Peripheral blood was collected regularly throughout the day, enabling day-long assessments of analytes. *N* = 8 per group; means ± SEM are depicted.

**Figure 2:**
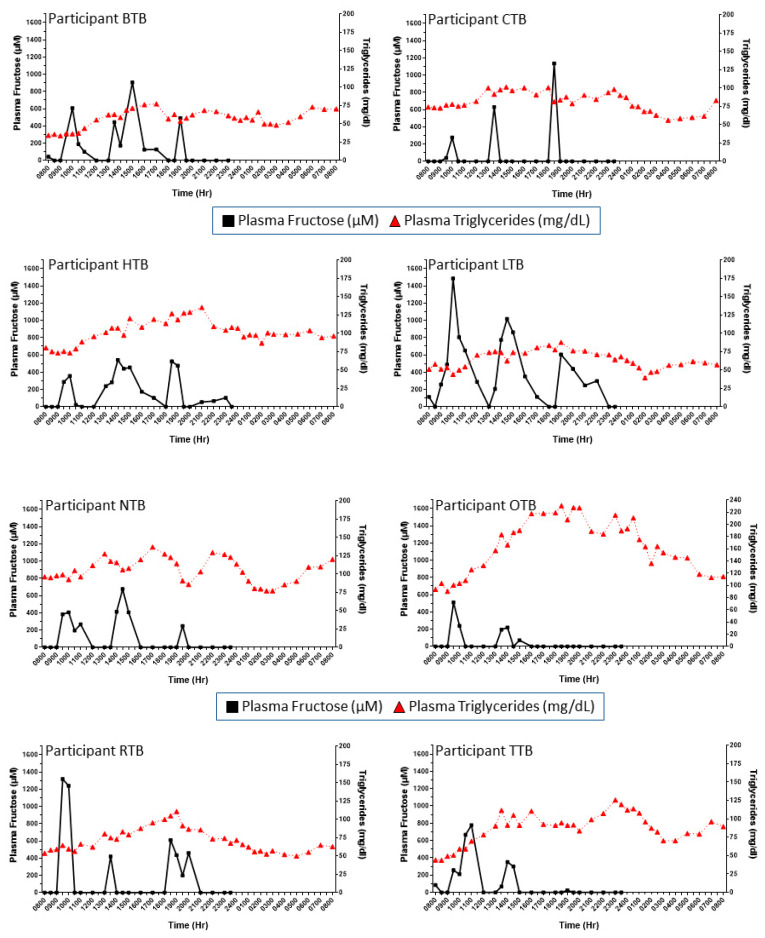
The substantial person-to-person differences in day-long and postprandial patterns of plasma fructose (squares, solid lines) and triglyceride (triangle, dotted lines) concentrations in women with obesity consuming breakfast, lunch, and dinner (each including a fructose-sweetened beverage). Each figure represents a single participant in Cohort 1.

**Figure 3:**
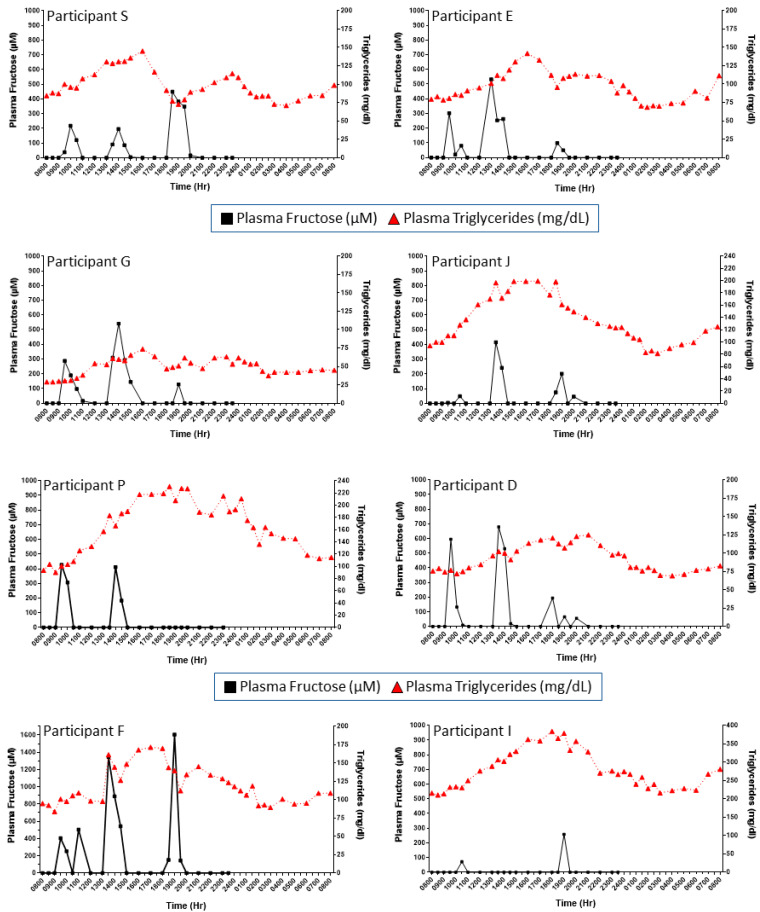
The substantial person-to-person differences in day-long and postprandial patterns of plasma fructose (squares, solid lines) and triglyceride (triangle, dotted lines) concentrations in men with obesity consuming breakfast, lunch, and dinner (each including a fructose-sweetened beverage). Each figure represents a single participant in Cohort 1.

**Cohort 1, primary study:** Considering the apparent sex differences in de novo lipogenesis in response to fructose consumption [36,37], we evaluated women and men separately. Figure 1 presents the grouped mean concentration patterns of plasma fructose in women (Figure 1A) and men (Figure 1B) studied throughout the day and following breakfast, lunch, and dinner meals that included fructose-sweetened beverages (see the Methods Section and Teff et al. [32]). To gain a sense of person-to-person fructose responses, the results in each subject were plotted separately. This approach revealed substantial interindividual differences in plasma fructose excursions following the meals (Figure 2 and Figure 3). Each person displayed unique patterns in terms of fructose concentration peaks, peak times, and peak patterns. For example, when looking at patterns in women (Figure 2), female participant BTB had her highest level of fructose after lunch, in contrast to female participant CTB’s highest fructose excursion occurring at roughly 1800 h, associated with when she consumed dinner. When comparing female participant CTB to female participant LTB, there is a marked difference in the peak levels of fructose in the systemic circulation as the day progressed: female participant LTB had decreases in peak plasma fructose throughout the day when comparing the three mealtimes. On the contrary, female participant CTB’s highest levels of postprandial plasma fructose increased as the day progressed. There were also remarkable differences in concentration peaks and timing patterns in the male subjects (Figure 3). For example, when comparing male participant S with male participant E, subject S had the highest peak of plasma fructose after dinner, whereas subject E exhibited the highest excursion after lunch, with a relatively small plasma fructose excursion following dinner. A survey of Figure 2 and Figure 3 also highlights the person-to-person variability in day-long plasma triglyceride levels (see triangle symbols within figures).

**Cohort 2, secondary study:** Plasma fructose concentrations were presented as log-transformed means in the original publication by Shen et al. [33]. The participants in the study consumed a low-fructose-containing beverage containing sugars, fats, and proteins, and fructose was detected with an MS-based metabolomics assay (values are semi-quantitative peak heights from mass spec). To explore fructose variability in more detail, we present non-transformed fructose data as means ± SD and also provide the results plotted individually. One participant (S13) exhibited a fructose pattern that was a clear outlier since their levels were extremely high at time zero (142660 and 142553, respectively) and dropped precipitously by 60, 120, and 240 min (to 19965, 13690, and 12727, respectively). It is speculated that this was due to prior consumption of a sugar-sweetened beverage or snack, and this participant’s results are not included for the result analyses below.

**Figure 4:**
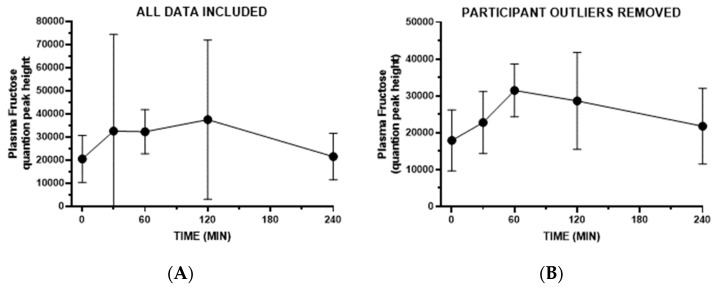
Postprandial plasma fructose concentrations in adults following the consumption of a liquid mixed macronutrient beverage containing a low amount of fructose (Cohort 2). (**A**) Mean ± SD for all participant data (*N* = 16) and (**B**) mean ± SD when excluding putative outlier participant data (see the Results Section: participants S1, S9, S28, and S34).

**Figure 5:**
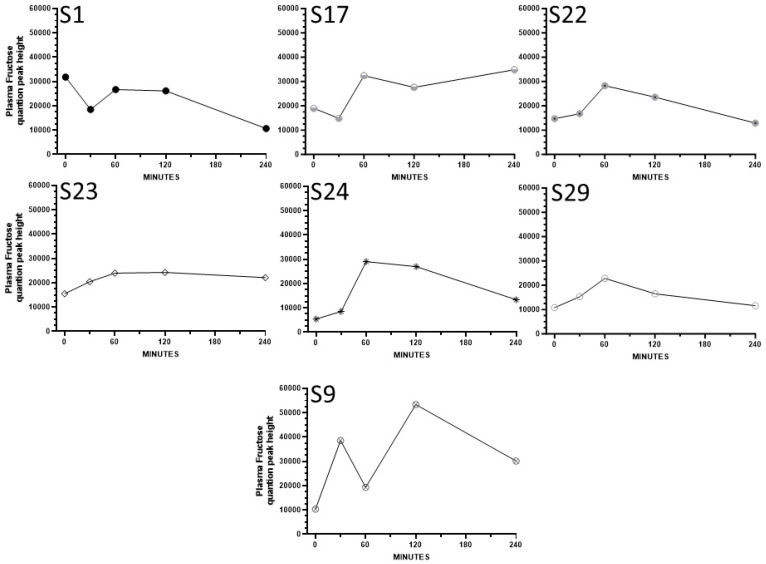
The substantial person-to-person differences in postprandial patterns of plasma fructose concentrations in women who consumed a mixed macronutrient drink containing a low level of fructose. Each figure represents a single participant in Cohort 2.

**Figure 6:**
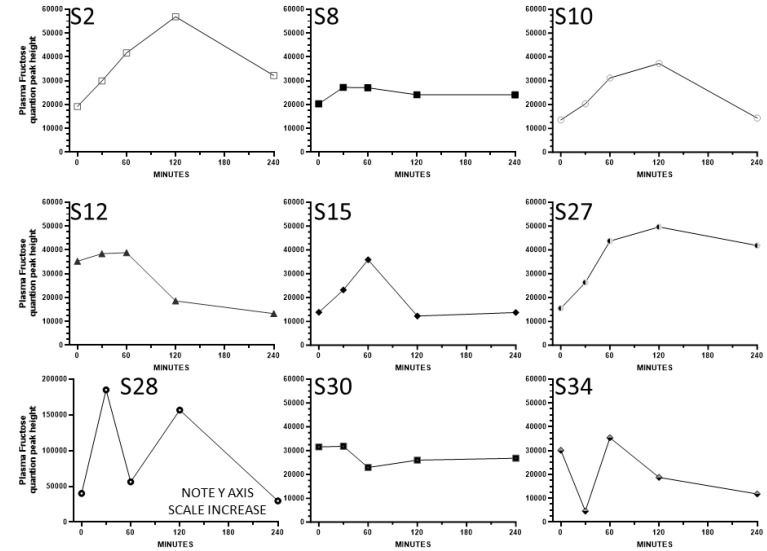
The substantial person-to-person differences in postprandial patterns of plasma fructose concentrations in men who consumed a mixed macronutrient drink containing a low level of fructose. Each figure represents a single participant in Cohort 2.

First, we evaluated cohort variance as a group. Figure 4A (left panel) depicts the mean ± SD for included participants, highlighting substantial variance, especially at the 30 min and 120 min time points (time effect, not significant). Exploratory analyses were conducted to consider if potentially anomalous timepoint data were impacting variability in fructose excursions. An examination of individual patterns (Figure 5 and Figure 6) revealed that the time zero value for participant S1 was higher than the 30 min value, and in the case of participants S9, S28, and S34, there were unanticipated single-timepoint decreases in plasma fructose at either 30 or 60 min. For exploratory purposes, we plotted the results without potential outliers S1, S9, S28, and S34. Overall variability dropped, and under this scenario, there was a clear increase in plasma fructose over the first 60 min (Figure 4B, right panel) (time effect, *p* = 0.009). Taken together, these results and the previously presented log-transformed data from all participants [33] indicate that the consumption of a low-fructose mixed macronutrient drink can elicit a rise in plasma fructose concentration in most individuals, and that responses can be highly variable.

Figure 5 and Figure 6 illustrate the short-term post-consumption plasma fructose concentration curves plotted for each female and male participant, respectively. A subset of participants exhibited relatively high postprandial increases in plasma fructose (i.e., S28), while others had smaller but detectable increases (i.e., S2, S9, S10, S15, S17, S24, S27), and several had small increases (i.e., S8, S22, S23, S29) or essentially no increase or erratic patterns (i.e., S1, S12, S30, S34). In some cases, over the 240 min sampling period, plasma fructose concentrations continued to increase, in contrast to other participants in whom fructose declined following the peaks. In some participants, the rise in fructose concentration occurred only after a lag time. Overall, the results under the low fructose intake conditions of the Cohort 2 study are consistent with the idea that there is significant person-to-person variability in postprandial circulating fructose concentrations and fructose peak patterns.

## 4. Discussion

There has been extensive research regarding the interindividual variability in postprandial metabolism of nutrients and food components, with glucose being the most prominent example. Fructose is also very prominent in the typical American diet, and excessive fructose intake has been implicated in cardiometabolic disease risk. Despite the prominence of this dietary sugar and its relevance to health, surprisingly little is known about interindividual differences in postprandial fructose metabolism and circulating fructose levels, or how these might be associated with metabolic health or disease risk. The purpose of this study was to address, for the first time, the person-to-person variation in postprandial fructose patterns in the systemic circulation under conditions of a mixed macronutrient challenge. The results highlight that across individuals, there are substantial differences in fructose concentration peaks and timing following intake of fructose-containing beverages consumed at a high dose with meals or following low-dose consumption via a mixed macronutrient beverage.

Postprandial plasma fructose concentrations can be impacted by a variety of physiological processes that vary across individuals. For instance, interindividual differences in gastric emptying, gastrointestinal [GI] motility, GI fructose transport and uptake from the lumen, or intestinal fructose metabolism could play a role. In the intestine, fructose is taken up most robustly when in the presence of glucose [38,39,40,41]; notably, the experimental paradigms of the current studies included the consumption of fructose with food sources of glucose. As described by Ferraris et al. [11], the process of fructose absorption in the intestines is largely driven by the transporter GLUT5. GLUT5 acts to transport fructose on the apical side of the cell, while GLUT2 transports fructose across the basolateral membrane. Ketohexokinase (KHK) is responsible for converting fructose to fructose-1-phosphate immediately following intestinal fructose absorption by GLUT5. GLUT5 might act as a transceptor [11], meaning that as the amount of fructose in the intestines increases, GLUT5 expression increases along with other important enzymes that contribute to the metabolism of fructose. It is interesting to speculate that differences in habitual consumption of fructose-containing foods could differentially modify the expression and activity of GI transporters and metabolic enzymes, which could, in theory, regulate the net uptake of fructose into the portal circulation. At least one study examined duodenal brush border membrane vesicle GLUT5 protein expression in a small number of individuals and demonstrated a very wide range within controls and within a group of subjects with type 2 diabetes, with a 4-fold higher mean level in the latter [42]. These observations support that interindividual variability in small intestine fructose absorption could contribute to disparate postprandial plasma fructose patterns. As for gastric emptying, it is well known that significant interindividual variability exists in this parameter; as one illustration, in a mixed-meal scintigraphy study of 80 patients with type 2 diabetes, impaired fasting glucose, or normal glucose tolerance, the gastric emptying half-life had a range from ~50 min to ~100 min [43]. Gut transit time, measured by tracing a blue orally administered dye in 800+ individuals from the PREDICT study, also revealed a wide range from ~0.5 days to ~2 days [44]. Although intestinal transporter activities, gastric emptying, or GI motility were not monitored in the current studies, they cannot be excluded as possible contributors to the person-to-person variation that was observed for postprandial plasma fructose peaks and patterns.

Tissue uptake and metabolism of fructose in the liver, kidney, adipose, and other tissues may also play a role in modifying postprandial plasma fructose excursions. For instance, the steady-state plasma fructose concentration was much lower (~2 mM) than that of glucose (~6–7 mM) when fructose was infused intravenously at the same rate as glucose (15 mg/kg/min) in rhesus macaques [34], similar to an earlier sugar infusion study in humans in which plasma fructose increases were just ~25% that of glucose [45]. Oral intake of equal amounts of fructose or glucose resulted in lower plasma concentration increases for the former in at least one study [46]. These outcomes support that whole-body tissue uptake of plasma fructose is robust and differs in mechanism from that of glucose. The metabolism of fructose has a unique relationship with triglyceride metabolism since catabolism of fructose yields significant substrates for de novo lipogenesis in the liver and intestines in humans [9,36,46]. Gluconeogenesis and metabolism to CO_2_ and lactate were also significant fates for oral ^13^C-fructose [9,37,46]. The liver plays a central role in these processes, since hepatic extraction of fructose has been estimated to be 50–80% in rats [13,15,18,19], ~65–80% in baboons [14], and ~70–80%+ in humans [16,17,20]. Thus, a viable hypothesis is that lower plasma fructose postprandial levels in some individuals could be explained in part by a more robust liver uptake and metabolism to end-products such as triglycerides, when compared to people with higher peripheral postprandial fructose concentrations. Validation of the plasma fructose–TG associations await larger-scale experiments specifically focused on tracking postprandial plasma fructose kinetics and hepatic uptake, comparing these to de novo lipogenesis rates. 

Another possible contributor to individual variability in postprandial plasma fructose patterns is the kidney. By monitoring arterial and renal vein concentrations of fructose during an intravenous infusion of fructose in healthy men, Björkman and Felig estimated that ~20% of infused fructose is absorbed and processed by the kidney [23]. Enzymes in the kidney can convert fructose to glucose, and the kidney expresses GLUT5, GLUT2, and SGLT5 transporters that can regulate the reabsorption of fructose from the descending loops (reviewed in [47,48]). In rats, kidney GLUT5 transporter expression varied based on prior exposures to fructose: the consumption of higher levels of fructose or sucrose increased the amount of GLUT5 by 3- to 4-fold [49]. Thus, individual variability in the plasma levels of fructose might in part be a result of varying activities of renal fructose filtration and kidney metabolism.

Finally, it is possible that white adipose tissue (WAT) and skeletal muscle contribute to the modulation of the plasma fructose pool. Adipocytes can take up fructose via glucose transporter 5 (GLUT5) [25,27,50]. Fructose uptake, fructose-associated glycerol synthesis, fructose combustion, and fructose conversion to lipids were evident in isolated rat adipocytes at >50–80% that of glucose [26,50], and like glucose, fructose can elicit leptin release from isolated adipocytes [28]. The potential quantitative contribution of skeletal muscle in whole-body fructose metabolism remains open to debate [24]. In a 4 h sugar infusion study in adults, supraphysiological plasma fructose concentrations (to ~5 mM) led to a net increase in muscle glycogen content [45]. In human muscle preparations ex vivo, fructose was readily taken up and metabolized to lactate in a dose-responsive manner [51], and in human skeletal muscle sarcolemmal vesicles (which express GLUT5), fructose uptake occurred but at a lower rate than glucose and via a distinct pathway [52,53]. The extent to which adipose or muscle fructose uptake and metabolism contribute to person-to-person differences in postprandial plasma fructose concentrations remains to be determined.

### Limitations, Implications, and Further Research

Sucrose and high-fructose corn syrups are widely used ingredients and sweeteners that are prominent in dietary patterns in many countries. The cardiometabolic disease risk thresholds for intakes of fructose or added sugars are potentially variable. Because of this, it is important to establish a better understanding of how dietary fructose is processed in the body and to determine the person-to-person variability in parameters reflective of fructose kinetics and metabolism, including hepatosplanchnic and body-wide postprandial exposures. The current study illustrates the substantial individual variations in the magnitude and timing of postprandial plasma fructose excursions following mixed macronutrient challenges containing low or high amounts of the sugar in adults. There are several limitations to our analyses and interpretations, and the current post hoc analyses should be considered preliminary findings. First, participant numbers were relatively small, and the results are observational in nature; thus, statistical associations between fructose AUC and phenotype aspects such as metabolic health status or metabolic flux measures await future study. For instance, correlation statistics comparing components of blood fructose patterns to rates of lipogenesis, liver or adipose insulin sensitivity, or adiposity would be of interest, using larger groups of participants that display large ranges of these parameters. Second, the analyses for the two cohorts utilized different assay platforms to measure plasma fructose concentrations: a quantitative biochemical assay with more limited sensitivity for Cohort 1 and a highly sensitive but only semi-quantitative metabolomics-based MS/MS determination of fructose for Cohort 2. The latter method detected fructose even in the fasted state, but this was not unexpected. As reviewed by Andres-Hernando et al. [54], endogenously produced fructose is produced naturally through the polyol pathway and is readily observed by mass spectrometry in human metabolomics experiments (i.e., [55,56]) or in studies using tissue ^1^H magnetic resonance spectroscopy scanning (i.e., [57]). This raises an interesting interpretive caveat in postprandial studies: a glucose challenge can lead to increased flux through the sorbitol pathway, which modestly increases plasma fructose concentrations measured by MS [55]. Third, it should be acknowledged that variance results can differ depending on the experimental paradigm and analysis method. For example, several studies have measured postprandial plasma fructose by mass spectrometry following the consumption of an equimolar glucose/fructose beverage or an HFCS beverage [58,59,60]; the results from two of these papers [58,60] report less variance in postprandial plasma fructose concentrations when compared with the results reported here, whereas the data presented in a 2018 report by Debray et al. indicate significant group variance [59].

Future research should focus on the dynamics and quantification of fructose uptake and metabolism in specific tissues including the liver, kidney, WAT, and muscle (and possibly erythrocytes; see [61]), and should consider how outcomes change over dose ranges typical of sugar intakes in the population. The mode of sugar delivery is also an important consideration. Fructose administered along with mixed meals or as part of a mixed macronutrient drink provide food matrices that engage complex digestive, endocrine, and metabolic systems. Additional studies are warranted to determine if interindividual variability in fructose digestion, metabolism, and postprandial blood concentration patterns is associated with cardiometabolic health phenotypes, disease risk, and digestive or liver functions. If these factors relate to one another, the development of fructose tolerance tests or continuous fructose monitoring could be envisioned in support of precision nutrition strategies or clinical care.

## Data Availability

Data associated with the study may be requested from the authors.

## Abbreviations

AUC	area under the curve
BMI	body mass index
GLUT	glucose transporter
KHK	ketohexokinase
MS	mass spectrometry
NAFLD	nonalcoholic fatty liver disease
SGLT	sodium–glucose linked transporter
